# Imaging of activated complement using ultrasmall superparamagnetic iron oxide particles (USPIO) - conjugated vectors: an *in vivo in utero* non-invasive method to predict placental insufficiency and abnormal fetal brain development

**DOI:** 10.1038/mp.2014.110

**Published:** 2014-09-23

**Authors:** G Girardi, J Fraser, R Lennen, R Vontell, M Jansen, G Hutchison

**Affiliations:** 1 MRC Centre for Inflammation Research, Queen's Medical Research Institute, University of Edinburgh, Edinburgh, UK; 2 Lupus Research Unit, The Rayne Institute, King's College London St Thomas' Hospital, London, UK; 3 Centre for Nano Safety, Napier University Edinburgh, Edinburgh, UK; 4 BHF/University Centre for Cardiovascular Science, University of Edinburgh, Edinburgh, UK; 5 Centrer for the Developing Brain, Division of Imaging Sciences and Biomedical Engineering, The Rayne Institute, King's College London, St Thomas' Hospital, London, UK

## Abstract

In the current study, we have developed a magnetic resonance imaging-based method for non-invasive detection of complement activation in placenta and foetal brain *in vivo in utero*. Using this method, we found that anti-complement C3-targeted ultrasmall superparamagnetic iron oxide (USPIO) nanoparticles bind within the inflamed placenta and foetal brain cortical tissue, causing a shortening of the T2* relaxation time. We used two mouse models of pregnancy complications: a mouse model of obstetrics antiphospholipid syndrome (APS) and a mouse model of preterm birth (PTB). We found that detection of C3 deposition in the placenta in the APS model was associated with placental insufficiency characterised by increased oxidative stress, decreased vascular endothelial growth factor and placental growth factor levels and intrauterine growth restriction. We also found that foetal brain C3 deposition was associated with cortical axonal cytoarchitecture disruption and increased neurodegeneration in the mouse model of APS and in the PTB model. In the APS model, foetuses that showed increased C3 in their brains additionally expressed anxiety-related behaviour after birth. Importantly, USPIO did not affect pregnancy outcomes and liver function in the mother and the offspring, suggesting that this method may be useful for detecting complement activation *in vivo in utero* and predicting placental insufficiency and abnormal foetal neurodevelopment that leads to neuropsychiatric disorders.

## Introduction

Identification of new biomarkers to predict bad pregnancy/foetal outcomes would be of enormous clinical benefit. In particular, a non-invasive detection of a biomarker that can identify pregnancies at risk would allow determining optimal timing of delivery and implementation of neuroprotective strategies to the mother. Currently, there are very few methods to predict adverse pregnancy/foetal outcomes.

Bad pregnancy outcomes have been associated with the activation of inflammatory pathways,^1^ in particular the complement system.^2,3^ Pregnancy complications such as antiphospholipid (aPL) antibody-induced placental pathology and foetal growth restriction and preterm birth (PTB) has been associated with complement activation.^4, 5, 6, 7^ Complement activation has also been related to abnormal development of the foetal brain cortex in premature birth in mice.^8^ In addition, a spectrum of brain abnormalities and cognitive impairment has been described in infants born to mothers affected by antiphospholipid syndrome (APS).^9, 10, 11^

During complement activation, C3 activation fragments (C3b/iC3b/C3d) are covalently attached to the injured tissue. Ultrasmall superparamagnetic iron oxide (USPIO, diameter: 5–40 nm) particles shorten the T2 and T2* relaxation time and are therefore used as negative contrast agents in magnetic resonance imaging (MRI).^12^ Moreover, by conjugating USPIOs to vectors that bind specific molecules, the USPIO can be used to detect those molecular targets *in vivo.* In the current study, we used USPIO nanoparticles conjugated to antibodies to C3 activation products as contrast agent of MRI to determine the presence of complement activation/inflammation in the placenta and foetal brain to predict pregnancy/foetal outcomes.

## Materials and Methods

### Animals

All housing and experimental procedures were performed in compliance with the UK Home Office Animals Scientific Procedures Act 1986 (Home Office project licence number 60/4305). C57BL/6 mice (2–3-month old) purchased from commercial vendors were used in all experiments. Females were mated with previously isolated males. The presence of a vaginal plug defined day 0 of pregnancy.

#### Mouse model of PTB (PTB model)

On day 15 of pregnancy, mice received intravaginal lipopolysaccharide to induce PTB.^4,8^ Mice were scanned in the morning of day 16. Scanned mice showed signs of preterm labor (vaginal bleeding and increased expression of connexin 43—marker of myometrial contractility—in myometrial biopsies postmortem). An age-matched group was studied as control. Mice were killed after imaging, the foetuses were weighed and the foetal brains were harvested for immunohistochemical (IHC) studies.

#### Mouse model of obstetric APS (APS model)

On days 8 and 12 of pregnancy, mice were treated with intraperitoneal injections of mouse aPL monoclonal antibodies (FB1, 1 mg). The control group received mouse immunoglobulin g (IgG; intraperitoneal, 1 mg). Mouse monoclonal aPL antibodies FB1 (IgG2bκ)—with anticardiolipin specificities and complement-binding capacity^13^—were obtained from NZW × BXSB F1 mice and generously provided by M Monestier (Temple University School of Medicine, Philadelphia, PA, USA).^13^

On day 15, the mice were subjected to MRI studies and killed after imaging. Placentas and foetal brains were collected for IHC and biochemical studies.

Placentas from the APS group and respective control group were analysed for oxidative stress levels and vascular endothelial growth factor (VEGF) content. Isoprostane 8-iso-prostaglandin F2a (signal transducer and activator of transcription factor 8 (STAT-8)) was measured as a marker for oxidative stress. Placental tissue was homogenised in nine volumes of 0.1 M Tris (pH 7.4) containing 1 mM EDTA and 10 μM indomethacin and stored at −80 °C in the presence of butylated hydroxytoluene (5 mg per 100 ml) before being assayed for free 8-isoprostane using a STAT-8-Isoprostane EIA kit (Cayman Chemical, Ann Arbor, MI, USA). For VEGF content measurements, placentas were homogenised in nine volumes of 0.1 M Tris (pH 7.4). Placental VEGF levels were measured by enzyme-linked immunosorbent assay (R&D Systems, Inc., Minneapolis, MN, USA). Placental growth factor (PlGF) was measured by IHC using a specific antibody anti-PlGF (Novus Biologicals, Littleton, CO, USA).

A group of control and APS pregnant mice were allowed to give birth, and behavioural studies were performed in the male offspring.

### Behavioural tests

Behavioural tests were performed in two male pups/mother 60 days after birth (PND60). Elevated plus maze and open-field tests were used to measure anxiety levels. In the open-field test, time spent in the centre and number of entries to the centre were recorded. In the elevated plus maze, time spent in the open arm and number of entries to the open arm were calculated. Behavioural tests were not performed in the PTB model, because the pups did not survive due to immaturity (day 16).

### MRI studies

#### Conjugation of anti-C3-antibodies with nanoparticules and test for C3 binding

Nanomag-D-SPIO 20 nm nanoparticles (surface COOH) and MACS separator with MS columns were purchased from Micromod (Miltenyi Biotech GmbH, Germany). USPIO were conjugated to monoclonal antibodies (rat IgG1) that bind specific complement activation C3 fragments C3b, iC3b and C3c deposited on tissue (clone 2/11 provided by John D Lambris, University of Pennsylvania, Philadelphia, PA, USA).^14^ The conjugation was performed following an established protocol that uses *N*-ethyl-*N*-(3-dimethyl aminopropyl) carbodiimide hydrochloride and *N*-hydroxy succinimide.^15^ Rat IgG1, isotype control antibody, was also conjugated to nanoparticles following the same protocol. The unconjugated antibodies were separated from conjugated antibodies by MACS column. The final products of conjugation were suspensions without precipitate. The amount of iron conjugated was determined by potassium thiocyanate method using FeCl3-6H2O as a reference.^16^

The presence of anti-C3 antibodies or isotype control antibodies on the surface of conjugated USPIO was confirmed by measuring protein content by Bradford methodand the amount of antibody in all preparations was 32–40 μg mg^−1^ nanoparticles.

The functionality/binding of USPIO-conjugated anti-C3 antibodies was confirmed by IHC. MRL/lpr mice develop lupus nephritis with deposition of C3 activation fragments in the kidney.^17^ Kidney frozen sections from MRL/lpr mice^17^ and C3−/− mice (negative control) were incubated with USPIO-conjugated anti-C3 antibody (dilution 1:100) and unconjugated anti-C3 antibody (dilution 1:100). A secondary antibody fluorescein isothiocyanate (FITC)-conjugated (Santa Cruz Biotechnology, Inc., Dallas, TX, USA, dilution 1:400) was used to develop the IHC reaction.

#### MRI protocols

Animals from the APS and PTB model groups and respective controls (mIgG-treated mice and untreated age-matched controls) received the USPIO particles (0.5 mg per mouse) (unconjugated, conjugated with anti-C3 antibody and conjugated with rat IgG1) intravenously (volume <100 μl) as a bolus (administration time, 2–3 s) 12–14 h before the imaging session. After the imaging sessions, mice were killed, and placentas and foetal brains were isolated for IHC and biochemical studies.

All MRI experiments were performed using a 7-Tesla horizontal bore Nuclear Magnetic Resonance spectrometer (Agilent Technologies, Yarnton, UK), equipped with a high-performance gradient insert (60 mm inner diameter), maximum gradient strength 1000 mT m^−1^. The mice were anesthetised with 1.8% isofluorane in oxygen/air (50/50, 1 litre min^−1^) and placed in a cradle (Rapid Biomedical GmbH, Rimpar, Germany). The rectal temperature and respiration rate were monitored throughout the experiments, and body temperature was maintained at 37 °C with a heat fan. A 33-mm diameter birdcage volume coil (Rapid Biomedical GmbH) was used for radio frequency transmission and signal reception. For anatomical assessment, respiration-gated T2-weighted fast spin echo images (echo train length of 8) of 1-mm slice thickness in a sagittal orientation were collected with the following parameters: repetition time (TR)≈3000 ms depending on the respiration rate; effective echo time=48 ms; 24 slices, field of view=35 × 35 mm^2^; matrix=192 × 192, 2 signal averages. For T2* mapping and calculation of T2* relaxation times, image acquisition used a gradient echo, multiple echo, untriggered pulse sequence of 10 images weighted in T2* TE=1.38, 2.94, 4.50, 6.06, 7.62, 9.18, 10.74, 12.30, 13.86 and 15.42 ms; TR=100 ms. From the anatomical images corresponding sagittal slices encompassing the most representative anatomies of interest (foetal brain, placenta, liver, muscle and kidney) were selected with a 35 × 35 mm^2^ field of view containing a 192 × 192 acquisition matrix (in-plane resolution=0.182 × 0.182 mm^2^). Slice thickness was 1 mm with 14 signal averages.

#### MRI data analysis

All images were processed and T2* maps generated with the VnmrJ software (Version 3.2A, Agilent Technologies). For each T2* map, regions of interest were drawn in each organ of interest making sure to stay well within the boundaries of the respective organ and the mean regions of interest T2* values were used as quantitative measures of T2* relaxation times (ms) for each tissue type.

### IHC studies

Foetal brains were harvested in the APS and PTB models and respective controls and incubated overnight in paraformaldehyde 4% with 10% sucrose for cytoprotection. Foetal brain tissue was then frozen in Optimal Cutting Temperature compound and cut into 10-μm sections. IHC studies were performed to evaluate the expression of microtubule associated protein-2 (MAP-2; Sigma Aldrich (St Louis, MO, USA) dilution 1/100)—a marker of intact neuronal cell bodies—and neurofilament 200 (NF-200; Sigma Aldrich, dilution 1/400)—to evaluate dendritic and axons structure. Neurodegeneration was measured using Fluoro-Jade B (Millipore, Millerica, MA, USA), a polyanionic fluorescein derivative which sensitively and specifically binds to degenerating neurons.^18^ Ten views per slide were analysed in each experimental condition.

Mice from the PTB and APS groups and respective controls that did not receive USPIO injections were killed after MRI, and placentas and foetal brains were processed for IHC. Frozen sections were stained for complement deposition, using monoclonal anti-C3b, -iC3b and -C3c antibodies (clone 2/11 provided by John D Lambris, University of Pennsylvania).^14^

To study whether USPIO-anti-C3 antibodies cross the placenta and the foetal blood–brain barrier (BBB), IHC studies were performed. FITC-conjugated antibodies to rat IgG1 were used to visualise the presence of USPIO-anti-C3 antibodies in the placentas and foetal brains in APS and PTB mice after the MRI studies. IHC studies to detect the presence of aPL antibody FB1 were also performed in the placentas and foetal brains from APS mice. PlGF was determined by IHC in frozen sections from placentas in APS and mouseIgG-treated mice. Confocal double-labelled microphotographs were captured using a Leica Microsystem (Leica, Milton Keynes, UK) with settings appropriate to the fluorophores present.

### Isolation of foetal cortical neurons

Cortical neurons from day 16 foetuses were isolated as previously described^8,19^ and cultured on coverslips coated with laminin/polylysin (5 × 10^5^ cell cm^−2^). Control cells extend their neurites and establish synapses in culture after 10 days in culture and thus represent an accessible model to study cortical brain development.^19^ After 7 days in culture (7 DIV), neurons were exposed to aPL antibody FB1 or control antibody (mouse IgG). Formation of neuritic networks in each experimental group was evaluated on 10 DIV by IHC using βIII tubulin antibodies after fixation with paraformaldehyde 4%.

### Effects of USPIOs on pregnancy

To study the potential effects of USPIOs in pregnancy, foetal and placental weight at day 16 was recorded in control and USPIO-treated mice after MRI imaging. Placental VEGF and isoprostane 8-iso-prostaglandin F2a STAT-8 content were measured by enzyme-linked immunosorbent assay as described above. Another group of control and USPIO-treated mice were allowed to give birth and time of delivery was recorded. To investigate clearance of USPIO nanoparticles in these groups, T2* maps for the liver were generated. Liver function tests were performed by measuring hepatic enzymes alanine transaminase (ALT) and aspartate transaminase (AST) with commercial kits (Abcam, Cambridge, UK) in the mother during and after pregnancy and in the offspring.

### Statistical analysis

Data are expressed as mean±s.d. Statistical differences between groups were determined using one-way analysis of variance with subsequent two-tailed Student's *t*-test.

## Results

### Conjugation of USPIOs to anti-C3 antibodies does not affect antibody-binding capacity

**Figure 1:**
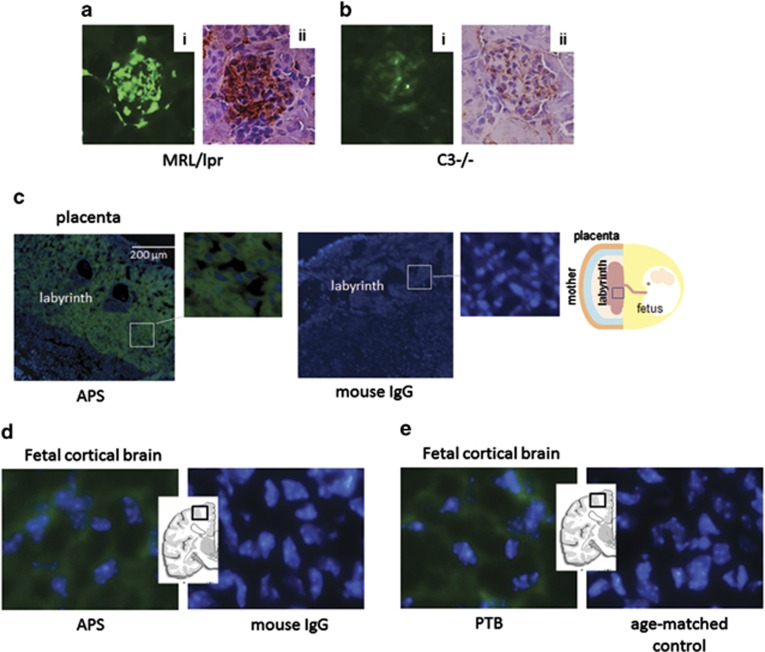
Binding capacity of ultrasmall superparamagnetic iron oxides (USPIOs) conjugated to anti-C3 antibodies compared with unconjugated antibodies. Passage of USPIO-conjugated antibodies through the placenta and foetal blood–brain barrier. (**ai**) USPIO-conjugated anti-C3 antibodies were used to detect C3 deposition in renal glomeruli from MRL/lpr mice. USPIO-conjugated anti-C3 antibodies were used as primary antibodies and fluorescein isothiocyanate (FITC)-conjugated as secondary antibodies. Positive staining was found in the kidneys from MRL/lpr mice. (**aii**) Detection of C3 deposition in the kidneys from MRL/lpr mice using unlabelled anti-C3 antibodies. (**bi**) USPIO-conjugated anti-C3 antibodies did not bind to the kidneys from C3-deficient mice (**bii**) Immunohistochemical studies using unlabelled anti-C3 antibodies demonstrate the absence of C3 deposition in renal glomeruli from C3-deficient mice (C3−/−), indicating that the binding of USPIO-conjugated anti-C3 antibodies to C3 is specific. (**c**) Immunohistochemical studies, using FITC-conjugated anti-rat immunoglobulin G1 (IgG1) performed after the magnetic resonance imaging studies show the presence of USPIO-conjugated antiC3 antibodies in the placental labyrinth of antiphospholipid syndrome (APS) mice (**c**), foetal brain from APS mice (**d**) and in foetal brain from preterm birth mice injected with USPIO-anti-C3 (**e**). The respective controls are mIgG-treated mice and age-matched mice. The first and third panels (left to right) in panel **c** show the placenta in APS- and mIgG-treated mice. Original magnification × 40. The second and fourth panels correspond to the labyrinth area. Original magnification × 20. Microphotographs (**a**–**e**) represent one of 4–5 similar experiments. The brain diagram shows the area of the cortical brain where the microphotographs were taken. The placental diagram shows the location of the labyrinth within the mouse placenta.

After conjugation, the reactivity of anti-C3 antibodies was verified by IHC. USPIO-conjugated anti-C3 antibodies were used to detect C3 deposition in glomeruli from MRL/lpr mice (Figure 1ai) and compared with unlabelled anti-C3 antibody (Figure 1aii). Using USPIO-conjugated anti-C3 antibodies as primary antibodies and FITC-conjugated secondary antibodies, C3 deposition was detected in the kidneys from MRL/lpr mice. USPIO-conjugated anti-C3 antibodies detect C3 (Figure 1ai) in a similar manner to unconjugated anti-C3 antibodies (Figure 1aii), indicating that the binding capacity of the antibody is preserved. In addition, USPIO-conjugated anti-C3 antibodies did not bind to the kidneys from C3-deficient mice (Figure 1bi) that do not express C3, as shown by IHC using unconjugated anti-C3 (Figure 1bii), indicating that the binding of USPIO-conjugated anti-C3 antibodies to C3 is specific.

### USPIO-conjugated anti-C3 antibodies cross the placenta and the foetal BBB

IHC studies performed after the MRI studies showed the presence of USPIO-conjugated anti-C3 antibodies in the placenta of APS mice (Figure 1c) and in the foetal brain of APS (Figure 1d) and PTB mice injected with USPIO-anti-C3 (Figure 1e) compared with their respective controls (mIgG-treated mice and age-matched controls). These data demonstrate that anti-C3 antibodies conjugated to USPIO cross the placenta and the foetal BBB. Within the foetal brain and placenta, most of the USPIO-conjugated anti-C3 antibodies were found in the cortex and the labyrinth, respectively.

### Injection of USPIO-conjugated anti-C3 caused a significant reduction in T2* relaxation time in foetal brains in PTB and APS mice indicating increased C3 deposition

**Figure 2:**
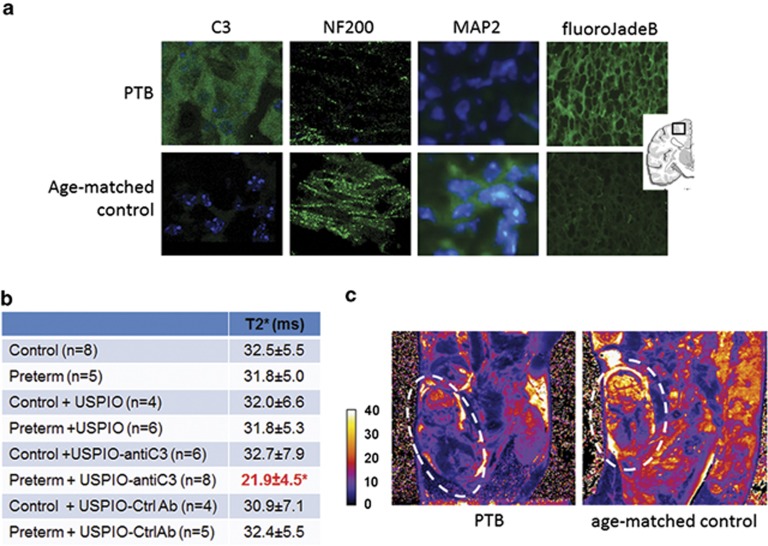
Increased C3 deposition, significant reduction in T2* relaxation time and abnormal foetal cortical brain cytoarchitecture in preterm birth (PTB) mice. (**a**) Confocal microscope photomicrographs demonstrating increased C3 deposition, decreased NF-200 and MAP-2 staining and increased neurodegeneration in the foetal brains in PTB mice compared with age-matched control. Microphotographs represent one of the five similar experiments. The brain diagram shows the area of the cortical brain where the microphotographs were taken. (**b**) Significant reduction in T2* time was observed *in vivo* in the foetal brains *in utero* in PTB mice treated with ultrasmall superparamagnetic iron oxide (USPIO)-anti-C3 compared with age-matched control and PTB mice that received USPIOs or control Ab-USPIOs. *Statistically significant compared with control mice injected with USPIO-anti-C3 (*P*<0.05), to preterm mice that received USPIO (*P*<0.05) and SPIO-Ctrl antibody (*P*<0.05) and preterm untreated mice (*P*<0.05). (**c**) T2* maps of maternal abdomen in a PTB mouse and age-matched control injected with USPIO-anti-C3 antibodies. The dashed ovals show the amniotic sacs containing the foetuses. Microphotographs represent one of 4–5 similar experiments.

We previously demonstrated abnormal cortical cytoarchitecture and increased neurodegeneration in the foetal brain in the mouse model of PTB.^8^ Cortical foetal brain abnormalities were associated with increased complement split product C5a levels. Interestingly, here we found increased C3 deposition in the foetal cortical brain in PTB mice (Figure 2a) compared with age-matched control. A significant reduction in T2* time—indicative of positive binding of USPIO-conjugated anti-C3—was observed *in vivo* in the foetal brains *in utero* in PTB mice compared with age-matched control and PTB mice that received USPIOs or control Ab-USPIOs (Figure 2b). Macrophages, present in inflamed tissue, may phagocytose USPIOs and thus cause a T2* signal diminution. T2* time in the placentas in APS and the foetal brains in APS and PTB mice injected with USPIOs was not different from USPIO-treated control mice, indicating that phagocytic cells do not have a significant role in the decreased T2* times observed in APS and PTB mice treated with USPIO-conjugated anti-C3-treated antibodies. Thus the MRI data suggest that the T2* signal diminution in APS and PTB mice is due to the presence of C3. T2* maps are shown in Figure 2c. As previously described, diminished NF-200 and MAP-2 staining was also observed in the cortex of foetal brains in PTB mice compared with age-matched control (Figure 2a), indicative of a disruption of cortical dendritic and axonal cytoarchitecture. In addition, increased neurodegeneration, measured by FluoroJade B, was also observed in the foetal cortical brain in PTB mice compared with age-matched control (Figure 2a).

**Figure 3:**
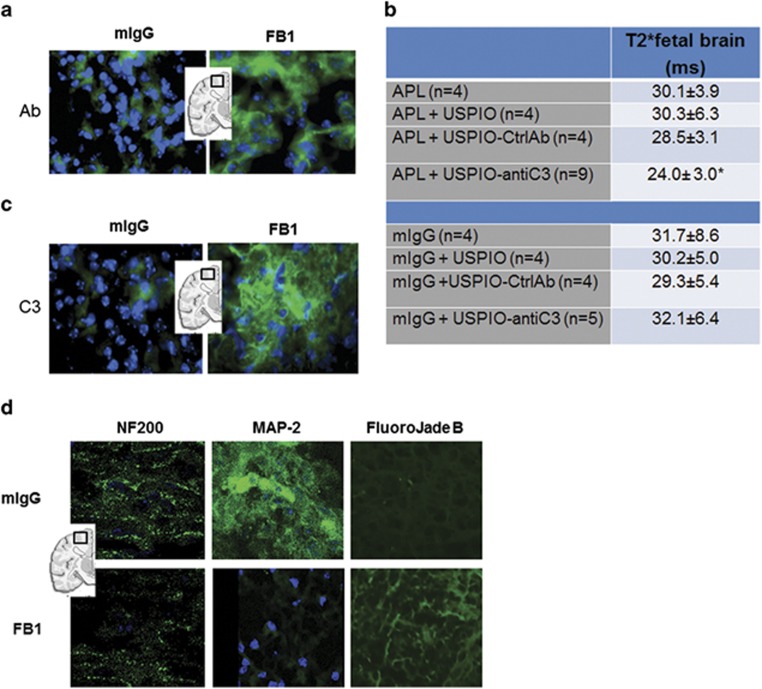
Antiphospholipid (aPL) antibodies bind to foetal brain. Increased C3 deposition, significant reduction in T2* relaxation time and abnormal foetal cortical brain cytoarchitecture in antiphospholipid syndrome (APS) mice. (**a**) Immunohistochemical studies demonstrating positive staining for aPL antibody FB1 in foetal brain tissue from APS mice. Microphotographs represent one of four similar experiments. The brain diagram shows the area of the cortical brain where the microphotographs were taken. (**b**) T2* values in APS mice that received ultrasmall superparamagnetic iron oxide (USPIO)-anti-C3. Asterisk (*): T2* values were statistically significantly lower in APS mice injected with USPIO-anti-C3 compared with APS mice that received only USPIO or mouse immunoglobulin G (IgG)-treated mice that received USPIO-anti-C3 (*P*<0.05 for all the comparisons). (**c**) Confocal microscope photomicrographs demonstrating the presence of increased C3 deposition in foetal brains in APS mice compared with mIgG-treated control mice. Microphotographs represent one of five similar experiments. The brain diagram shows the area of the cortical brain where the microphotographs were taken. (**d**) Confocal microscope photomicrographs demonstrating NF200 and MAP-2 staining in the foetal brains in APS mice compared with mouse-IgG treated. Diminished NF200 and MAP-2 staining suggest an abnormal neuronal development. Increased neurodegeneration is also observed in foetal brains in APS mice. Microphotographs represent one of fivesimilar experiments. The brain diagram shows the area of the cortical brain where the microphotographs were taken.

A similar association between complement deposition and abnormal cortical foetal brain development was observed in the APS model. First, we demonstrated that aPL antibodies FB1 are able to cross the BBB and reach the foetal brain. Indeed, FB1 antibodies were found in the foetal brain cortex by IHC (Figure 3a). T2* values in APS mice that received USPIO-anti-C3 were lower than APS mice that received only USPIO or mouse IgG-treated mice that received USPIO-anti-C3 (Figure 3b). This suggests that complement C3 deposition is observed in the foetal brains in the APS model. IHC studies confirmed the presence of C3 deposition in the foetal brain cortex in APS mice (Figure 3c). Similar to the PTB, increased C3 deposition and abnormal cortical brain development were observed in the brains of foetuses exposed to aPL *in utero*. The diminished NF200 and MAP-2 staining observed in the foetal brains in APS compared with mouse-IgG treated mice suggest an abnormal cortical neuron development (Figure 3d). In addition, increased neurodegeneration was observed in the foetal brains from APS mothers (Figure 3d).

### Neurotoxic effects of aPL antibodies

**Figure 4:**
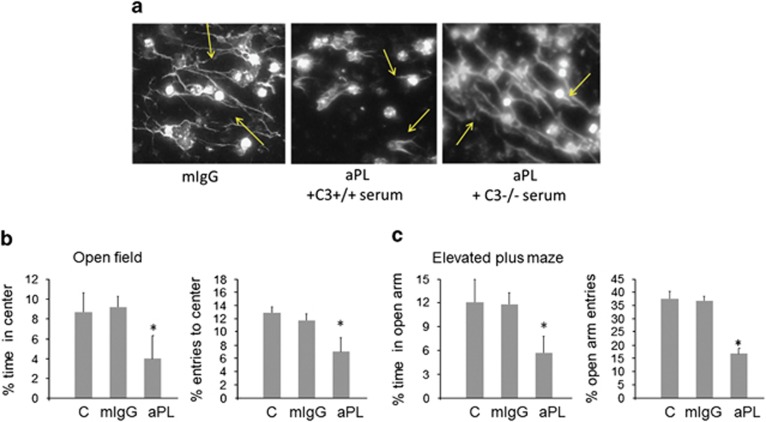
Antiphospholipid (aPL) antibody FB1 induces neurotoxicity *in vitro*. Behavioural studies in the offspring of antiphospholipid syndrome (APS) mice. (**a**) Neuritic network formation in isolated foetal cortical neurons incubated with aPL and mouse immunoglobulin G (IgG). Incubation with aPL antibodies induced a significant decrease in total length of b-III-tubulin-positive neurites, compared with untreated neuronal cells, suggesting abnormal development of cortical neurons. In the absence of complement component C3 (serum from C3-deficient mice), aPL did not affect neuritic network formation. Cells were identified as neurons by positive BIII tubulin staining. Five to six cortical neurons preparations were used for each experimental condition. (**b**) Offspring from APS show a significant decrease in the percentage of time spent in the centre and a significant reduction in the number of entries into the centre in the open-field test when compared with control group (*P<0.05) and mice born to mouse IgG-treated mothers (*P<0.05) consistent with anxiety-related behaviour. Five to six mice were studied in each experimental group. (**c**) In the elevated plus maze, offspring from APS mothers showed a significant decrease in the number of times they explore the open arms and the time spent in the open arms was also less when compared with mice born from control (*P<0.05) and mIgG-treated mothers (*P<0.05). Locomotor activity did not change among groups. Five to six mice were studied in each experimental group.

To confirm the detrimental effects of aPL on the developing foetal brain, we studied isolated cortical neurons from day 16 foetuses. IHC procedures determined that most cultured cells (>95%) express βIII tubulin (Figure 4a), protein associated with cortical neurons and not found in glial cells. These cells extend their neurites and establish synapses becoming mature neurons after 10 days in culture. To study the effects of aPL on developing neurons *in vitro*, we exposed the cells on day 7 (7 DIV) to FB1 antibody. The neuronal culture media includes foetal calf serum as a source of complement. As a quantitative measure of neuronal injury during neuronal development, we analysed the growth of projections emanated from the neuronal cell bodies. At day 10, long axons were observed in cultured control cortical neurons (incubated with mouse IgG) (162±32 μm), whereas the length of axons in aPL-exposed neurons was considerably reduced after the addition of aPL to the media on 7 DIV (39±11 μm) (Figure 4a). Incubation of cortical neurons with aPL in the absence of complement C3 (C3−/− serum) prevented aPL-induced neurotoxic effects. Long axons were observed in cultured cortical neurons incubated with FB1 plus C3-deficient serum, suggesting a role for complement in aPL-induced neurotoxicity.

### Behavioural abnormalities in the offspring in the APS model

The open-field test and the elevated plus maze test showed increased anxiety-related behaviour in the offspring from APS mice (Figures 4b and c). Offspring from APS mothers show a significant decrease in the percentage of time spent in the centre and a significant reduction in the number of entries into the centre in the open-field test (Figure 4b) when compared with control group and mice born to mouse IgG-treated mothers) consistent with anxiety-related behaviour. In the elevated plus maze, offspring from APS mothers showed a significant decrease in the number of times they explore the open arms and the time spent in the open arms was also less when compared with mice born form control and mIgG-treated mothers (Figure 4c). Locomotor activity did not change among groups.

### Decreased T2* relaxation time in placentas from APS mice that received anti-C3-USPIO

**Figure 5:**
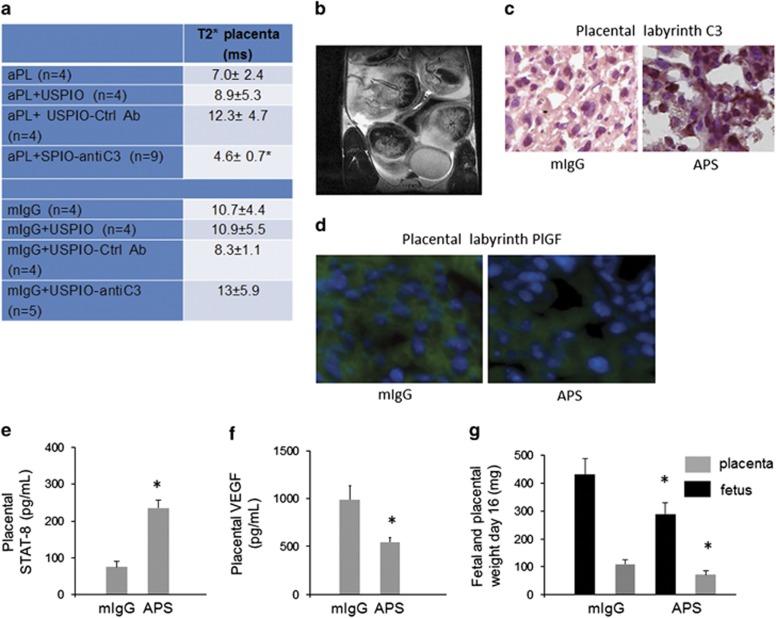
Decreased T2* relaxation time in placentas from antiphospholipid syndrome (APS) mice that received anti-C3-USPIO (ultrasmall superparamagnetic iron oxide). (**a**) Decreased T2* time was observed in placentas from APS mice that received USPIO-anti-C3 antibodies compared with untreated APS mice (*P<0.05), APS mice treated with USPIOs alone (*P<0.05), APS mice injected with USPIO-Ctrl AB (*P<0.05) and control immunoglobulin G (IgG)-treated mice injected with USPIO-anti-C3 (*P<0.05). (**b**) Fast pin echo magnetic resonance imaging coronal view showing a placenta in APS mice. (**c**) Increased C3 deposition in placentas from APS mice compared with control mIgG-treated mice, detected by immunohistochemistry. Microphotographs represent one of five similar experiments. The placenta diagram shows the area of the labyrinth where the microphotographs were taken. (**d**) Increased oxidative stress—measured as signal transducer and activator of transcription factor 8 content in placentas from APS mice compared with mIgG-treated mice. *n*=4–5 mice/group. (**e**) Decreased levels of vascular endothelial growth factor in placentas from APS mice compared with mIgG-treated mice. *n*=4–5 mice/group. (**f**) Immunohistochemical detection of placental growth factor in placentas from APS- and mIgG-treated mice. (**g**) Intrauterine growth restriction and decreased placental weight in foetuses from APS mice compared with control mice treated with mouse IgG. *n*=4–5 mice/group.

Complement activation and placental inflammation are crucial events in obstetric APS.^20^ Decreased T2* time was observed in placentas from APS mice that received USPIO-anti-C3 antibodies compared with APS mice treated with USPIO alone and control mouse IgG-treated mice injected with USPIO-anti-C3 (Figure 5a). The fast pin echo MRI coronal view (Figure 5b) clearly shows a placenta in APS mice. Increased C3 deposition in placentas from APS mice was also detected by IHC (Figure 5c). Abundant C3 deposition was observed in the labyrinth, crucial placental structure responsible for gas and nutrient exchange between the mother and the foetus. Increased C3 deposition in placentas from APS mice was associated with increased oxidative stress—measured as STAT-8 content—(Figure 5d) and decreased levels of VEGF (Figure 5e) and PlGF (Figure 5f). In addition, intrauterine growth restriction and decreased placental weight were observed in foetuses from APS mice compared with control mice treated with mouse IgG (Figure 5g).

### USPIO administration did not affect liver function and pregnancy outcomes

**Figure 6:**
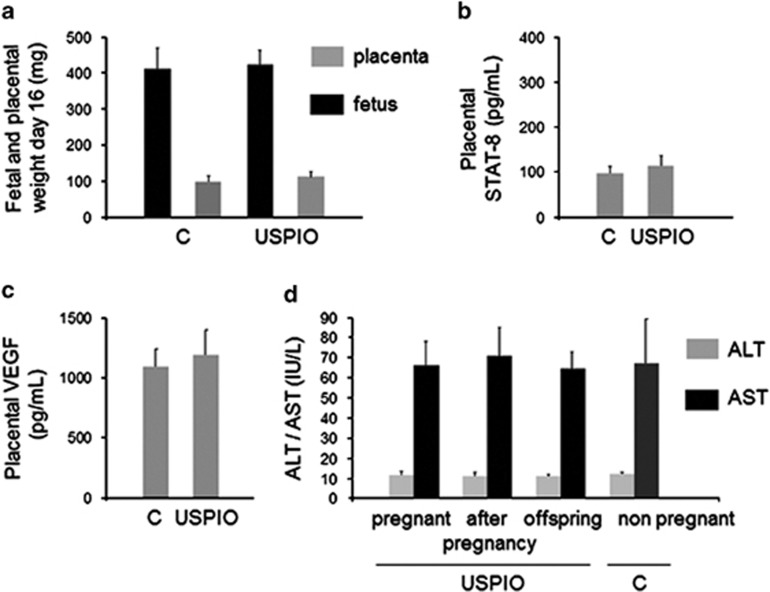
Effects of ultrasmall superparamagnetic iron oxide (USPIO) on pregnancy outcomes and liver function in the mother and offspring. (**a**) Foetal and placental weight at day 16 in USPIO-treated mice were not different from control untreated mice. *n*=4–5 mice/group. (**b**) Oxidative stress levels (signal transducer and activator of transcription factor 8 levels) in placentas from USPIO-treated mice were not different from control mice. *n*=4–5 mice/group. (**c**) Vascular endothelial growth factor levels in placentas from USPIO-treated mice were not different from control mice. *n*=4–5 mice/group. (**d**) Hepatic enzymes (alanine transaminase and aspartate transaminase) activity in the serum in mothers during pregnancy (–day 14) and postpartum (21 days) and offspring in USPIO-treated and control mice.

It has been described that administration of silica and titanium dioxide nanoparticles (0.8 mg per mouse, nanoparticle diameter: 35 and 70 nm) causes pregnancy complications.^21^ In the present study, mice treated with USPIO nanoparticles (0.5 mg per mouse, diameter: 20 nm) had normal pregnancy outcomes. USPIO-treated mice gave birth at term like control groups (pregnancy length (days): USPIO (*n*=4): 20.2±0.8 vs control (*n*=6): 20.6±0.5)). In addition, foetal and placental weights at day 16 in USPIO-treated mice were not different from control (Figure 6a). Oxidative stress levels (STAT-8 levels) and VEGF concentration in placentas from USPIO-treated mice were not different from control mice (Figures 6b and c). T2* values in placentas from control untreated-mice were similar to USPIO-treated mice (T2* (ms): control (*n*=6): 10.2±4.3 vs USPIO (*n*=4):9.1±5.7), suggesting that USPIO nanoparticles do not accumulate in the placenta. Interestingly, a lower T2* value was found in the maternal liver of USPIO-treated mice when compared with control (T2* (ms): control (*n*=6): 13.7±2.2 vs USPIO (*n*=4):4.4±2.6*, *P<0.01), suggesting that USPIOs are cleared by the liver. Measurement of hepatic enzymes AST and ALT activity showed that USPIOs do not affect liver function. AST and ALT activity during pregnancy and after pregnancy in USPIO-treated mice were not different from control females (Figure 6d).

## Discussion

PTB is a major public health problem.^22^ More than 15 million babies are born prematurely worldwide. When babies are born before they are fully developed, they face many serious medical problems, such as neurodevelopmental impairment.^23^ Unfortunately, in the majority of the cases of PTB, there are few tests that can be employed to predict if a foetus is at risk for the development of brain injury. In the APS, foetuses exposed to aPL antibodies *in utero* are also at risk. In APS, miscarriage, foetal death and placental pathologies are common.^20,24,25^ There is growing evidence about the transplacental passage of aPL antibodies, and a spectrum of brain abnormalities and cognitive impairment has been described in infants born to mothers affected by APS,^9, 10, 11^ suggesting that exposure to aPL *in utero* affects foetal brain development and thus might induce behavioural and cognitive problems later on in life. In our studies, we showed that, during pregnancy, complement-activating aPL antibody FB1 crosses the placenta and the BBB reaching the foetal brain and affecting its normal development. This is in agreement with the literature that shows that maternal antibodies have direct access to the developing brain during gestation.^26^

Inflammation, in particular complement activation, is a common mediator in the pathogenesis of PTB and obstetric APS.^2,4,5,7,27^ In this study, using nanotechnology, we successfully developed a non-invasive *in utero* method to determine the presence of complement activation/inflammation in the placenta and foetal brain to predict pregnancy/foetal outcomes in PTB and pregnancies complicated with APS.

USPIO particles are magnetic resonance contrast agents that give rise to a significant shortening of T2 and T2* relaxation time.^12^ USPIOs can be conjugated to vectors directed to specific targets and detected by MRI *in vivo*. In this study, we conjugated USPIOs to monoclonal antibodies to complement C3 split products (C3b, iC3b, C3c) to detect complement activation *in vivo in utero* by MRI. Importantly, we were able to show that USPIO-conjugated anti-C3 antibodies cross the placenta and the foetal BBB reaching the foetal brain. That IgG antibodies cross the placenta and the foetal BBB is in accordance with other studies.^28,29^ In addition, the BBB is developing during gestation allowing antibodies to have direct access to the brain during gestation.^30^

Injection of USPIO-conjugated anti-C3 caused a significant reduction in T2* relaxation time—indicative of increased C3 deposition—in the placentas and foetal brains from abnormal pregnancies. Increased C3 deposition in the placenta and foetal brains was associated with abnormal cortical foetal brain development and placental abnormalities in pregnancies compromised by PTB and APS. In the APS model, increased C3 deposition in placenta was associated with placental insufficiency characterised by increased oxidative stress, decreased levels of proangiogenic factor VEGF and PlGF and decreased placental and foetal weight. This suggests that measuring C3 deposition using USPIOs and MRI during pregnancy might be a useful method to predict placental pathologies, intrauterine growth restriction and foetal brain abnormalities.

Generally, T2 relaxation time diminishes when blood flow is diminished and oxyhaemoglobin levels are low. Considering the increased oxidative stress observed in placentas in APS mice, we expected to see a diminished T2 signal in untreated APS-mice compared with control mice. Although T2* signal values in placentas from APS mice were slightly lower than in mice treated with control antibody, they did not reach statistical significance. This data suggest that placental blood flow is not significantly compromised in APS.

C3 deposition in the foetal brain was associated with cytoarchitectural cortical neurons abnormalities in PTB and APS mice. We previously demonstrated that complement activation has a detrimental role in cortical brain development in preterm foetuses and *in vitro* using cultured isolated foetal cortical neurons.^8^ Here we also showed that the neurotoxic effects of aPL on neuritic network formation *in vitro* is also dependent on complement activation. The role of complement in brain injury remains controversial, and most of the previously published studies were performed in adult animals. In favour of the deleterious effects of complement in foetal brain injury, a study showed that C3 is depleted in the blood of neonates who subsequently develop cerebral palsy.^31^ In addition, complement depletion has shown to improve cerebral blood flow and neurological function after ischaemia in adult rats^32^ and reduced post ischaemic cerebral infarct volume and atrophy in neonatal rats.^33^ Other studies showed that complement inhibition failed to prevent brain injury caused by thromboembolic stroke^34^ and hypoxia–ischaemia^35^ in adult animals. A recent study showed that hypoxic/ischaemic injury in neonates is ameliorated in mice that overexpress C3a.^36^ However, in this study, hypoxic/ischaemic injury was induced at postnatal day 9, and only white matter injury was evaluated. Our studies demonstrate an association between increased foetal brain C3 deposition and abnormal cortical foetal brain development in day-16 foetuses *in utero*. Interestingly, we were able to correlate cortical brain abnormal development with behavioural changes in the APS mice. Increased anxiety was observed in the offspring of APS mice, suggesting that *in utero* exposure to aPL antibodies causes abnormal cortical development and suggests that C3 might be a footprint for adverse foetal outcomes. Our studies associating abnormal foetal cortical development with increased anxiety in the offspring of APS mice are in agreement with the literature that shows that, during prefrontal cortex brain injury, the neural networks involved in the regulation of anxiety are compromised.^37^ Interestingly, it has been described that adult mice with APS showed a pattern of moving along the edges of the open field rather than randomly through the centre.^38^ A similar behaviour was observed in the offspring of APS mothers in our studies.

Although C3 deposition in the foetal brains and placentas in APS can be simply explained by the activation of the classical pathways by aPL antibodies,^5,27^ the cause of C3 activation in the foetal brains in PTB is not fully understood. We previously proposed that C5a generated in the cervix during preterm labour can reach the *utero* and the foetal brain. We can speculate that C5a—generated during spontaneous preterm labour—could activate neurons to release reactive oxygen species, which further cleave C3 creating a complement activation feedback cycle.

It was previously demonstrated that placental complement deposition is associated with adverse pregnancy outcomes in APS in humans and mice.^20^ Interestingly, complement deposition in human placentas in APS was found in the villous tree, vascular bed site for feto-maternal exchange, equivalent to the labyrinth in mice.^20^ Here we show that increased placental complement deposition is predictive of placental insufficiency resulting in intrauterine growth restriction in APS.

A recent study also demonstrated the usefulness of superparamagnetic iron oxide-conjugated nanoparticles to detect complement activation *in vivo*.^39^ In this study, the authors successfully showed complement deposition in glomerulonephritis in mice by using SPIO particles conjugated to human complement receptor 2.^39^

In conclusion, using this *in utero* non-invasive method, we found that complement activation is a footprint for cortical foetal brain abnormalities and behavioural changes in pregnancy complicated by APS and of abnormal neuronal cytoarchitecture in the cortical brain in PTB. In addition, increased complement deposition in placentas predicted placental abnormalities and intrauterine growth restriction in the mouse model of APS. Contrary to a previous publication, showing that nanoparticles cause pregnancy complications,^18^ our data show that USPIO administration to pregnant mice is safe and no placental/foetal abnormalities are observed. The MRI studies show that the USPO nanoparticles used in this study—of smaller size than the ones used in the previous study—are rapidly cleared through the liver. Normal liver function was observed in USPIO-treated mothers (during pregnancy and after pregnancy) and in their offspring when compared with control animals, emphasizing that USPIO are not hepatotoxic. That USPIOs did not affect pregnancy outcomes suggests that this non-invasive *in utero* method to detect complement activation using anti-complement antibodies conjugated to USPIOs might be safe to use in humans and might help predict bad pregnancy/foetal outcomes in pregnancies at risk. The identification of foetuses with neurodevelopmental and/or placental abnormalities during pregnancy would allow the possibility of giving the mother neuroprotective strategies to modulate inflammation and prevent abnormal foetal autcomes.
